# A Report on the Influenza Epidemic in the British Armies in France, 1918

**Published:** 1919

**Authors:** 


**A Report on the Influenza Epidemic in the British Armies in France, 1918.** By the Influenza Committee of the Advisory Board to the D. G. M. S., France. *The British Medical Journal*, November 9, 1918.

The epidemic of influenza began by a few local outbreaks in the First and Second Armies in April and May.

It spread with great violence in the Second Army. The admissions to its casualty clearing stations were extremely numerous during the month of June. 3,851 cases were admitted during the seven days following June 1, and on June 25, 683 cases were admitted to clearing stations. By the middle of July the number of admissions was averaging 150 daily, and had fallen to about 50 daily by the middle of August.

In the first Army the total admissions for influenza between May 18 and July 2 were 36,473.

B. influenza had appeared frequently before in the armies and it is rather difficult to determine why the disease had not become epidemic before. It seems that the general health of the army is as good now as ever it was.

Some particular conditions, almost world-wide, have probably arisen, favoring the growth of the bacillus, since the disease has spread over all Europe, and there has been concomitant epidemic in the United States.

The disease has proved extremely infective, and this is a point of chief importance to distinguish influenza from trench fever.

*Incubation Period.* The period of incubation was from two to four days.

Two medical cases suffering from influenza were sent to a surgical ward of N° 55 General Hospital, and careful observation showed how the disease spread so as to infect the occupants of most of the beds near the two medical cases.

The epidemic had a very mild character in the garrison in the Calais area. Mortality averaged about 0.2 per cent. of the total number.

*Symptoms and Clinical Course of the Disease.* The onset was usually sudden. The initial symptoms were headache, pains in the back and limbs, and a feeling of weakness. Actual shin pains, which are common in trench fever, were seldom observed. Pains of a severe aching character lasted as a rule two or three days.

On an average, temperature rose to 102 and 103° F. This was reached usually on the first but occasionally on the second day. It fell generally by lysis and reached the normal on the sixth day. However, in a considerable number of very mild cases fever lasted only forty-eight hours, and convalescence was very short.

The pulse was rapid during the first two days, though usually under 120, and dilatation of the right heart occurred frequently as a result of pulmonary disease.

Catarrhal symptoms were seldom observed at the beginning of the epidemic, but later on pharyngitis and tracheitis were frequent. The serious complications which developed are discussed below. Several symptoms of less importance were met with. In some cases a rash occurred principally on the neck, the shoulders, the wrists and the dorsum of the feet, but no wheals were observed. Conjunctivitis and coryza were mentioned by several medical officers. In a few cases enlargement of the spleen occurred. The tongue was usually coated with a slight grey or white fur; loss of taste and of smell was common; and the disease began frequently by vomiting and tenesmus.

*Progress.* In the earlier stages of the epidemic, progress was rapid, and three days' incubation, three days' fever, and three days' convalescence was about the rule. As the intensity and the virulence of the epidemic increased, the pyrexial period was lengthened, and convalescence also.

*Relapses.* Occasionally a short relapse of fever and symptoms were observed about the end of the first week; this became more frequent in the later stages of the epidemic.

*Complications.* Severe complications, which at first had been very rare, occurred frequently at a later stage of the epidemic. They chiefly affected the lungs, and cases of bronchitis, broncho-pneumonia and lobar pneumonia were reported.

The cases of bronchitis and bronchopneumonia were severe, with a high and irregular temperature, a rapid pulse, dyspnoea and cyanosis. Many of them proved fatal.

When lobar pneumonia occurred, the fever was more regular, but it often fell by lysis. This complication was frequent, especially among the Chinese.

From June 25 to June 29, 1 700 cases were admitted into N° 1 Canadian Casualty Clearing Station; 42 of the patients suffered from bronchopneumonia and 3 from lobar pneumonia. Of these 45 patients, 4 died.

It was observed that in the First Army 1 per cent. of the cases developed bronchopneumonia, of which 10 to 15 per cent. died.

In 440 cases at a field ambulance, 22 per cent. suffered from a slight bronchitis; 9.7 per cent. from a moderate bronchiolitis; and 9 per cent. had to be evacuated for severe bronchitis, catarrhal or lobar pneumonia, and pleurisy.

The chief complications after the pulmonary ones were albuminuria and nephritis. In the beginning of the epidemic patients were completely free from albuminuria, but this complication seemed to appear concomitantly with pulmonary infection.

During the winter of 1916-17 several cases of nephritis were observed as a complication of bronchitis and bronchopneumonia. At one casualty clearing station in the Second Army 10 such cases proved fatal, and sections of the kidney led to the supposition that a renal lesion occurred previous to the influenzal attack.

Meningitis developed in a few cases. The B. influenzae was found in the meninges and in the spinal fluid. In the Second Army four such cases occurred, of whom three died; B. influenzae could not be recovered from the bacteriological cultures and smears.

*Diagnosis.* Influenza and trench fever present very similar symptoms. Relapses so frequent in trench fever are rather seldom in the present epidemic. Where many cases are observed simultaneously, it may be assumed that the disease is influenza. While in trench fever sequelae are almost always confined to the heart, pulmonary and renal complications occur in the latter disease.

*Treatment.* The treatment adopted has been purely symptomatic.

Attempts have been made to discover B. influenzae in the secretions from the respiratory tract, nasopharynx and the circulating blood.

*Methods Employed for the Isolation of B. Influenzae.* A loopful of pharyngeal secretion washed away from the fresh sputum was stroked upon a plate of nutrient agar containing blood. Excellent cultures of B. influenzae were obtained in this manner, by heating this agar slope to 80° C. B. influenzae was also recovered from citrated blood incubated for three days.

*Result.* In the earlier stages of the epidemic, bacteriologists succeeded very seldom in isolating B. influenzae. The studied sputum contained a great number of pneumococci, streptococci, and micrococcus catarrhalis, which probably exist normally in the respiratory tract but are not so numerous. Later on it was found much easier to recover B. influenzae from those cases in which definite catarrh of the respiratory tract had occurred. Bacteriologists who studied the muco-purulent discharge produced in the first days of the disease succeeded in 80 per cent. of their attempts.

When tracheitis, bronchitis or bronchopneumonia had developed, the cultivation of the sputum gave nearly pure cultures of B. influenzae.

*Morphology and Cultural Characters of the Bacillus Isolated.* The small bacillus found in most cases had all the characteristic features of Pfeiffer's bacillus influenzae. In direct smears of the sputum or lung it often occurred in patches. It was pleomorphic and its length varied from 0.7 to 1.2 $\mu$, while its breadth varied from 0.3 to 0.4 $\mu$. It stained well with dilute carbol fuchsin.

Although presence of blood was generally necessary for the growth of these bacilli, cultures sometimes succeeded with ordinary nutrient agar planted from sputum or naso-pharyngeal mucus, but not subculture.

The primary culture upon blood-agar from the sputum furnished, after twenty-four hours' incubation, hemispherical strains from 0.1 to 0.2 mm. in diameter. On subculture upon blood-agar they grew well and reached sometimes a diameter of 0.5 to 1 mm. The bacilli obtained in cultures were larger than those in the original sputum. Cultures isolated in France died out very soon and weekly subculture at least was necessary to maintain them.

*Virulence.* B. influenzae seems to possess little virulence for rabbits and rats.

*Summary of 30 Postmortem Examinations.* Obsolete pulmonary or glandular tubercle was present in 7, or 23 per cent.; chronic nephritis in 2; pleuritic adhesions in 12; bilateral 4, unilateral 8.

*Lung.* Pneumonia was absent in none.

| | |
|---|---|
| Bronchopneumonia was present in | 25 |
| Confluent pneumonia | 11 |
| Lobar pneumonia | 1 |
| Bronchiolitis or "miliary pneumonia" | 5 |
| Pulmonary abscess | 2 |
| Pulmonary collapse (2 with small effusions) | 5 |
| Marked emphysema | 2 |
| Purulent bronchitis | 14 |

*Pleura.*

| | |
|---|---|
| Recent pleurisy | 24 |
| Bilateral | 15 |
| Unilateral | 9 |
| Effusion | 10 |
| Subpleural or interstitial hemorrhages | 18 |
| Enlarged and inflamed bronchial glands | 15 |

*Heart.*

| | |
|---|---|
| Dilatation | 29 |
| Myocarditis | 21 |
| Endocarditis | 2 |
| Pericarditis | 0 |

*Spleen.* Average weight, 7 1/2 oz.; mostly soft and pale.

*Liver.* Inclined to fatty change; slight toxic jaundice in three cases.

*Kidney.* " Toxic nephritis " occurred in 10. Average weight 12 1/2 oz. the pair. One showed hyaline thrombosis of glomerular vessels.

*Brain.* Abscess was present in one case showing purulent bronchitis. Meningitis was not found.

*The Number and Distribution of the Leucocytes in the Blood.* The number varied between 6000 and 11000 per c. mm., and the distribution was within normal limits. That is why pathologists did not pursue their researches.

*Evidence of the Production of Antibodies to the Hemophilic Bacilli Isolated.* An attack of influenza may predispose to another, probably because some influenza bacilli remain in the upper respiratory tract and threaten to reinfect the organism at the first occasion.

Animals injected with dead cultures of influenza bacilli present a great quantity of agglutinins.

The sera of patients taken two to three weeks after the onset of the disease seemed nearly normal, whereas the sera examined four to ten days after the onset agglutinated a culture in dilutions of 1 in 80 to 1 in 320 in different cases; normal serum could not agglutinate the same emulsion in dilutions higher than 1 in 20.

Increased phagocytosis of the hemophilic bacilli occurs obviously as a response to the disease. The sputa of three cases suffering from bronchitis were examined daily and afforded pure cultures of small Gram-negative hemophilic bacilli. Until seventeen days after the onset of the disease the bacilli were extracellular; then sudden change was noticed and the majority of the leucocytes contained the bacilli often to the number of forty to fifty per cell. Active phagocytosis was observed as long as the sputum contained the bacilli, and improvement occurred at the same time in the patient's condition. Though it has not been ascertained, this phagocytic activity was accompanied by the development and outpouring of opsonins.

*Consideration of the Evidence Pointing to B. Influenzae as the Cause of the Epidemic.* Three reasons led to the supposition that Pfeiffer's bacillus was the etiological factor in the epidemic: (1) It was found frequently in the respiratory tract of patients suffering from catarrh; (2) It has been recovered from the blood of patients in a few severe cases; (3) It commonly occurs in the lesions of those cases who succumb to complications.

